# Treadmill-to-Overground Mapping of Marker Trajectory for Treadmill-Based Continuous Gait Analysis

**DOI:** 10.3390/s21030786

**Published:** 2021-01-25

**Authors:** Woo Chang Jung, Jung Keun Lee

**Affiliations:** Inertial Motion Capture Lab, School of ICT, Robotics & Mechanical Engineering, Hankyong National University, 327 Jungang-ro, Anseong 17579, Gyeonggi, Korea; chwch93@gmail.com

**Keywords:** treadmill-to-overground mapping, optical motion capture system, gait analysis

## Abstract

A treadmill was used to perform continuous walking tests in a limited space that can be covered by marker-based optical motion capture systems. Most treadmill-based gait data are analyzed based on gait cycle percentage. However, achieving continuous walking motion trajectories over time without time normalization is often required, even if tests are performed under treadmill walking conditions. This study presents a treadmill-to-overground mapping method of optical marker trajectories for treadmill-based continuous gait analysis, by adopting a simple concept of virtual origin. The position vector from the backward moving virtual origin to a targeted marker within a limited walking volume is the same as the position vector from the fixed origin to the forward moving marker over the ground. With the proposed method, it is possible (i) to observe the change in physical quantity visually during the treadmill walking, and (ii) to obtain overground-mapped gait data for evaluating the accuracy of the inertial-measurement-unit-based trajectory estimation. The accuracy of the proposed method was verified from various treadmill walking tests, which showed that the total travel displacement error rate was 0.32% on average.

## 1. Introduction

People usually walk 10,000 steps a day and close to hundreds of millions of steps throughout their lives [1]. This is why gait analysis is constantly being actively studied. Marker-based optical motion capture (MoCap) systems are considered a gold standard in biomechanical gait research due to their high accuracy [2,3,4,5,6,7,8,9,10,11,12]. In MoCap-based gait analysis, markers are attached to the lower limb of subjects and the gait is analyzed using the trajectories of these markers. However, MoCap systems have a limited capture volume, as their measurement is based on fixed cameras. Accordingly, in gait research using MoCap, the subject’s gait is limited to, at most, a dozen steps. Thus, gait data are usually obtained by repeating a fixed course within a limited space [6,7].

MoCap is often combined with a treadmill to achieve a continuous walking trajectory for biomechanical research in a limited space [13,14,15,16,17,18,19,20]. A treadmill provides a convenient experiment environment in terms of unlimited gait trackability under volume constraint and speed controllability. Although treadmills allow continuous walking, the MoCap data repeated in a limited space during the treadmill walking are overlapped in the restricted space. Therefore, most of the gait data obtained through treadmill walking are analyzed based on gait cycle percentage. That is, each gait period from one heel strike to the next heel strike of the same foot is the time normalized as one gait cycle and the average and standard deviation of a physical quantity with respect to the gait cycle are used for the gait analysis [5,6,7,13,14,15,16,17,21,22,23,24].

However, achieving continuous walking motion trajectories over time without time normalization is often required. It should be noticed that human gait is not consistent. For example, the stride length, cadence, and gait pattern of a pedestrian can meaningfully change during the measurement due to, for example, muscle fatigue. This is particularly true in the case of patients who experience discomfort in walking, such as stroke and Parkinson’s patients [15,16,22,23,24]. In this situation, the gait-cycle-based analysis may not be effective, as it is difficult to observe the change in physical quantity according to the movement in the forward direction such as overground walking. This is a motivation for treadmill-to-overground (T2OG) mapping of the gait data.

**Figure 1:**
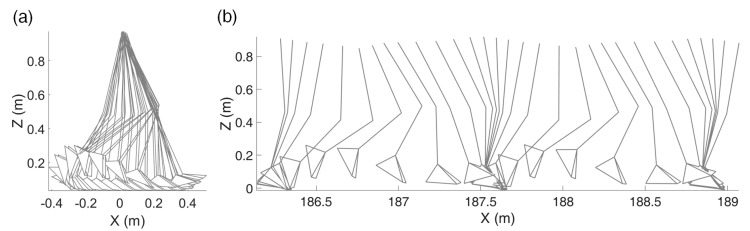
Examples of walking trajectory from each system during the same treadmill walking test: (**a**) trajectory from a marker-based optical motion capture (MoCap) system, (**b**) trajectory estimated using inertial measurement unit (IMU) signals.

More importantly, another motivation for the T2OG mapping originates from gait research using inertial measurement units (IMUs). Recently, IMUs have been actively used in gait analysis [14,21,25,26,27,28,29,30,31,32,33]. This is mainly because they can overcome the inherent in-the-lab limitation of optical MoCap systems in terms of measuring volume, due to their sourceless (or self-contained) property not requiring external sources [28,34,35]. Wearability of IMUs due to the sourceless property provides tremendous benefits that could be associated with long-term activity monitoring of patients and individuals in home and community settings [36,37,38]. The prerequisite for obtaining gait data through IMUs is IMU-based estimation of position or trajectory, which requires a process of double integration of gravity-compensated acceleration. It should be noticed that, even if the data measurements are performed on a treadmill, the position estimated through the double integration of the acceleration proceeds in the forward direction, like overground walking. Therefore, to verify the accuracy of IMU-based trajectory estimation at the research stage, a comparison with MoCap-based trajectory data, which are used as a reference, is required. For this, mapping of the MoCap-based trajectory data collected during the treadmill walking to overground is necessary. In other words, direct comparison of IMU-based trajectory and MoCap-based trajectory is not possible without the mapping process (see Figure 1).

With regards to the above two motivations, this study deals with the T2OG mapping of optical marker trajectories for continuous gait analysis. Using the proposed T2OG mapping, the marker trajectory during the treadmill walking on a track belt rotating in an infinite orbit is not confined to a limited treadmill walking volume but extends on an unfolded belt, which can be considered the overground.

The second section presents the T2OG mapping method and describes the experiments under various conditions for the validation of the method. The third section presents the results of experiments showing the accuracy of the T2OG mapping. Finally, several discussion points are made, and conclusions are drawn in the fourth section.

## 2. Method and Experiments

### 2.1. Treadmill-to-Overground Mapping Method

The basic concept of the proposed T2OG mapping method is as follows. As the treadmill belt moves backward, the position of the origin of a frame attached onto the belt moves backward, and this moved origin is called a virtual origin. The position vector from the virtual origin (which is “moving backward”) to a targeted marker (which is moving within a limited walking volume of the treadmill) is the same as the position vector from the “fixed” origin to the marker moving “forward” over the ground. Hence, the key process of the T2OG mapping is the estimation of the position (or displacement) of the virtual origin, which is the same as the estimation of the belt displacement change while moving backward.

**Figure 2:**
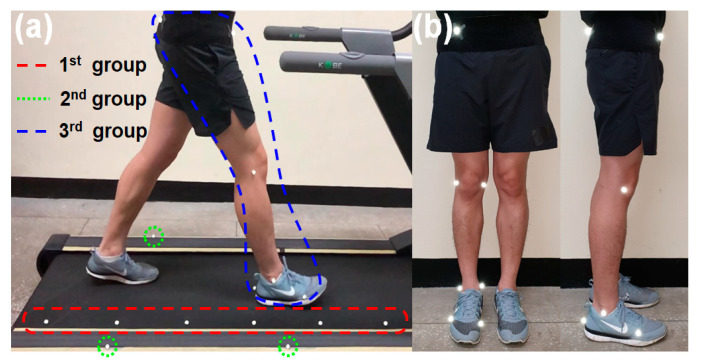
(**a**) Walking test setup with markers attached to a treadmill and a subject; (**b**) subject-attached markers viewed from the coronal and sagittal planes.

Three groups of markers of a MoCap system are attached for the mapping (see Figure 2a). The first group of markers is attached onto the treadmill belt in line. Hereafter, these markers are referred to as a “marker chain”. The marker chain is used to estimate the displacement of the virtual origin, which plays a key role in the mapping. At least two markers of the marker chain must be captured while the treadmill is operating. The total number of markers in the marker chain can vary depending on the length of the belt and other setting conditions. The second group of three markers is attached onto the side panel of the treadmill to form a treadmill frame {*TR*}. The treadmill frame is required to consider the inclination change of the treadmill. The third group of markers is attached to body segments to be tracked. 

**Figure 3:**
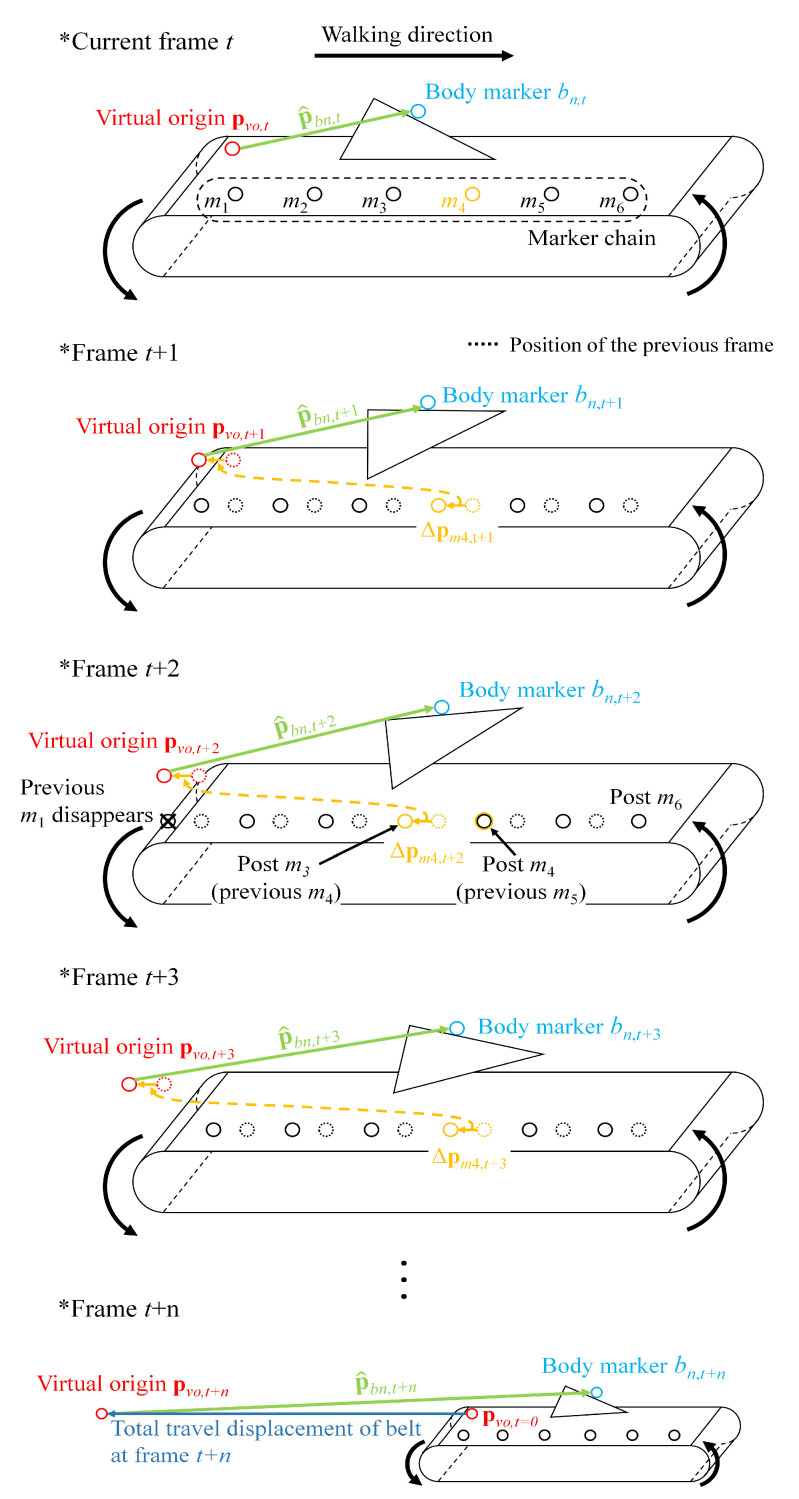
Mapping procedure over the time frame.

The captured marker of the marker chain is denoted as $m_{n}$ where the subscript *n* indicates the *n*th marker from the rear end of the treadmill (see Figure 3). Note that the markers of the marker chain attached onto the moving belt will appear and disappear, repeatedly. Therefore, shifting between a series of adjacent markers occurs. For instance, before the marker $m_{1}$ disappears under the running track, the next marker $m_{2}$ is shifted and designated as the post $m_{1}$, and the marker $m_{3}$ becomes the post $m_{2}$, sequentially. The position of the marker $m_{n}$ is denoted as $p_{mn}$ and its position change during one time step $\Delta p_{mn,\;t}$ is

(1) $$\Delta^{TR}p_{mn,\;t}{=}^{TR}p_{mn,\;t}{-}^{TR}p_{mn,\;t-1}$$

where the subscripts t and t−1 indicate the current and previous steps, respectively, and the superscript *TR* indicates that the corresponding vector is expressed with respect to the treadmill frame {*TR*}.

In T2OG mapping, it is essential to estimate the position of the virtual origin ${}^{G}p_{vo,\;t}$ which can be expressed as

(2) $${}^{G}p_{vo,\;t}={\;}^{G}p_{vo,\;t-1}+\Delta^{G}p_{vo,\;t}$$

where the position increment of the virtual origin with respect to the global frame {*G*}, $\Delta^{G}p_{vo,\;t}$, is nonetheless the position change of the belt during one time step. In Equation (2), as the movement of the marker chain is the same as the movement of the virtual origin, $\Delta^{G}p_{vo,\;t}$ can be replaced with $\Delta^{G}p_{mn,\;t}$. Note that any marker in the marker chain can be selected for $\Delta^{G}p_{mn,}$ as long as it is well captured and is thus suitable to represent the belt movement. In this study, the 4th marker $m_{4}$ is selected, as it is at the center of the capture volume and thus can be stably captured by the MoCap cameras. Subsequently, Equation (2) can be rewritten as

(3) Gpvo, t= Gpvo, t−1+RTRG ΔTRpm4, t

where RTRG is the orientation of {*TR*} with respect to {*G*} and is available via the second group of markers. At the moment of shifting, $p_{m4,\;t}$ and $p_{m4,\;t-1}$ are obtained from different markers. Accordingly, Δpm4, tTR at the shifting moment should be differently treated, i.e.,

(4) $$\Delta^{TR}p_{m4,\;t}^{}=\{\begin{array}{l} {}^{TR}p_{m4,\;t}{-}^{TR}p_{m4,\;t-1}\;,\;\;\;\mathrm{if}\;\;\;\left\|\Delta^{TR}p_{m4,\;t}\right\|<\varepsilon \\ {}^{TR}p_{m3,\;t}{-}^{TR}p_{m4,\;t-1}\;,\;\;\;\mathrm{otherwise}\; \end{array}$$

where ε is the threshold to determine the shifting moment based on the norm of $\Delta^{TR}p_{m4,\;t}$.

Next, the increment $\Delta^{TR}p_{m4,\;t}$ is projected onto the *x*-axis of the treadmill frame XTR to remove belt movement other than in the walking direction. This projection procedure is required to avoid error accumulation due to the unavoidable belt deflection caused by the weight or movement of the pedestrian. The projected $\Delta^{TR}p_{m4,\;t}$ is obtained as

(5) ΔTRpm4,proj,t=XTR(ΔTRpm4,t⋅XTR)

Consequently, the position of the virtual origin in Equation (3) is replaced by

(6) Gpvo, t=Gpvo, t−1+RTRG ΔGpm4,proj,t

Finally, when the *n*th marker attached to the body is denoted by $b_{n}$, the overground-mapped position of the body marker ${}^{G}{\hat{p}}_{bn,t}$ is estimated by subtracting the position of the virtual origin ${}^{G}p_{vo,t}$ from the body marker position pbn,tG.

(7) Gp^bn,t=pbn,tG− Gpvo, t

Figure 3 is an illustration of the T2OG mapping procedure. $\Delta p_{m4,\;t}$ is continuously updated to estimate the position of the virtual origin $p_{vo,t}$. The body marker position mapped to the overground ${\hat{p}}_{bn,t}$ is estimated by using the relative vector from the virtual origin to the body marker $p_{bn,t}$. The frame *t* + 2 in Figure 3 shows the shifting moment when $m_{1}$ disappears. 

### 2.2. Experiments

A treadmill KOBE-4000 (Kobesports, Goyang, Korea) and an optical motion capture camera system OptiTrack Flex13 (Natural Point, Corvallis, OR, USA) were used to verify the proposed T2OG mapping method. Seven cameras were used and the MoCap data were sampled at 120 Hz. In this study, the marker chain has 14 markers with intervals of 25 cm on a 350 cm-long belt. Six or more markers are captured on the belt during treadmill operation with this setting. In the treadmill setup, the time duration and speed were controlled with a treadmill built-in program and the inclination was adjusted manually. Two types of tests were conducted: one without pedestrian walking and the other with pedestrian walking.

#### 2.2.1. Test without Pedestrian Walking

The displacement of the treadmill belt represents the displacement of the pedestrian in the T2OG mapping result. Therefore, it is essential to estimate the displacement of the belt accurately for precise mapping. We conducted a test without pedestrian walking to verify the accuracy of the estimation of belt displacement without any influences due to pedestrian walking. The total travel displacements (TTDs) of the belt with respect to the frame {*TR*} obtained from the reference and the proposed method were compared. The reference of the belt displacement was estimated from the number of revolutions of the belt counted manually and the pre-measured length of the belt. In addition, the unmeasured extra length was considered. The TTD from the proposed method was obtained using ${}^{TR}p_{vo,x}$, which is the heading (or *x*-direction) component of ${}^{TR}p_{vo}$.

**Figure 4:**
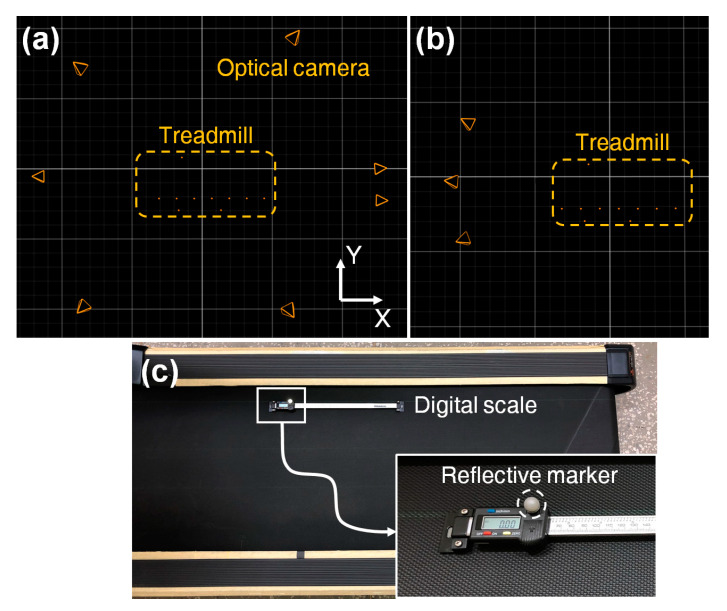
Setup for treadmill test without pedestrian walking: (**a**) camera setting 1, (**b**) camera setting 2, and (**c**) setup for evaluation using a digital linear scale.

Two different camera settings of the MoCap system were tested to evaluate the effect of camera settings on the estimation accuracy: setting 1 wherein seven cameras were evenly placed around the treadmill (see Figure 4a) and setting 2 wherein three cameras were placed only behind the treadmill (see Figure 4b). The tests were conducted for five minutes at three different speeds: 2, 4, and 6 km/h. Each test was repeated five times. Additionally, the accuracy of the MoCap system was evaluated using a digital linear scale, ME-HZ300 (Machine DRO, Hoddesdon, UK). The ruler had a resolution of 10 μm and a maximum operating range of 300 mm (see Figure 4c). This evaluation was conducted to identify the cause of errors when comparing the TTDs from the reference and the proposed methods. A reflective marker was attached onto the translating part of the digital linear scale, and then the part and the marker were translated by approximately 30 cm. The translating distances from the ruler and the MoCap system were compared. 

#### 2.2.2. Test with Pedestrian Walking

The pedestrian walking tests were conducted to demonstrate the T2OG mapping results. As a case study, one healthy male subject (age 27 years, height 176 cm, and weight 70 kg) performed two different tests: (i) constant-speed walking without inclination and (ii) variable-speed walking with various inclinations. The constant-speed walking test was performed at 4 km/h for five minutes. For the variable-speed walking test, which lasted for ten minutes, the applied speed and inclination were as follows: 4 km/h without inclination for three minutes → 1 km/h without inclination for three minutes → 4 km/h with the inclination of 4° for three minutes → 4 km/h without inclination for one minute. Each test was repeated three times, i.e., for three trials each. 

Nine markers were attached to the right lower limb and pelvis of the subject: three markers to the foot, two markers to the ankle, two markers to the knee, and two markers to the pelvis. The positions of the joints were estimated by averaging the two marker positions of the joints (see Figure 2b). The reference data for comparison were collected in the same way as in the previous test without the pedestrian test. The camera setting was also the same as the setting 1 of the previous test where cameras were evenly placed around the treadmill.

## 3. Results

Table 1 shows the estimation results of the TTD for the tests without pedestrian walking, in terms of averages and standard deviations for five trials. Table 1a shows the results of setting 1. In this setting, the error increased when the speed was increased. However, the TTD error rate remained almost constant regardless of the speed, i.e., 0.19% on average. In Table 1b, which is based on setting 2, although the magnitude of the error rate increased compared with that in setting 1, the error rate tended to be constant regardless of speed, i.e., 1.01% on average. Therefore, the proposed T2OG mapping method shows consistent performance for different treadmill speeds. Comparing a and b in Table 1, the TTD error rate increased by approximately 0.82% as the camera setting was changed from setting 1 to setting 2. Therefore, a proper camera setting is required to ensure the estimation accuracy. The error rates from the linear scale were 0.16 ± 0.004% for setting 1 and 0.91 ± 0.003% for setting 2. The error rate increased by approximately 0.76% as the camera setting was changed from setting 1 to setting 2. This shows that the TTD error rates shown in Table 1 and Table 2 are close to the error rates from the linear scale. Consequently, it can be said that most of the errors in the T2OG mapping are caused by an accumulation of the errors from the MoCap system.

Table 2 shows the TTD estimation results for the tests with pedestrian walking. Table 2a, which presents the results of the constant-speed walking test, shows an error rate of 0.32%, which is higher than the error rate of 0.18% for the test without pedestrian walking at 4 km/h. The error rate from the linear scale of the walking test was 0.28%, which shows only a difference of 0.04% from the TTD error rate. Note that the camera setting has not been changed between the two tests but the MoCap system calibration has been renewed for each test. Therefore, it can be concluded that the estimation accuracy of belt displacement was not significantly affected by pedestrian walking. Instead, a slight difference caused by the calibration process resulted in an increase in the error rate. Table 2b shows the belt TTD estimation results for walking tests under various conditions. The constant-speed and variable-speed walking tests have the same camera arrangement and initial calibration. Consequently, the average TTD error rates of the two tests showed a difference of only 0.01%. The proposed T2OG mapping method shows consistent performance regardless of the walking conditions.

**Figure 5:**
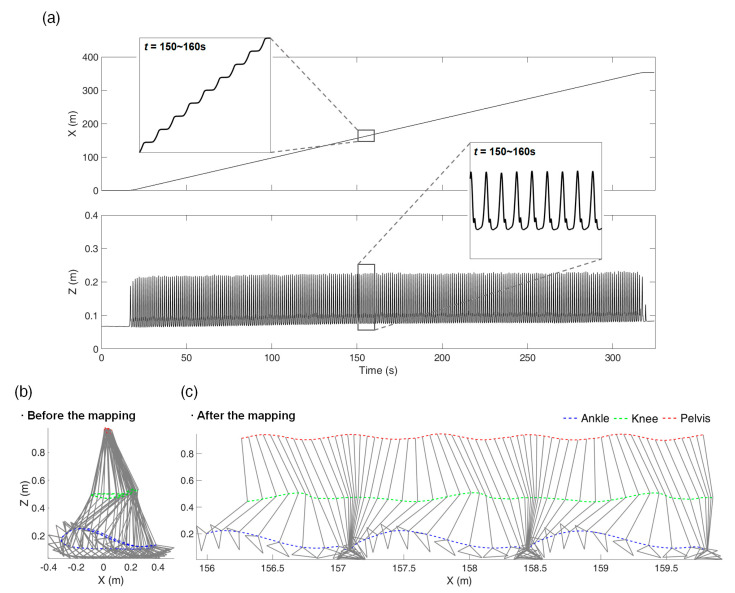
Mapping results from Trial 3 of the constant-speed test without inclination: (**a**) shows the *x*- and *z*-axes ankle marker trajectories with respect to time, (**b**,**c**) show marker trajectories on the sagittal plane before and after the mapping, respectively (*t* = 150–153 s).

Figure 5 is the mapping result of Trial 3 with the constant-speed walking test as an example. Figure 5a shows the *x*-axis (heading direction) and *z*-axis (vertical direction) trajectory mapping results of the ankle marker over time, and Figure 5b shows the marker trajectories on the *x*–*z* plane before and after the overground mapping during the time range of 150–153 s. The marker trajectories before the mapping show repetitive movements within the range of 0.8 m and the pelvis marker shows little movement. After the mapping, as the marker trajectories spread to the overground, the stance and swing phases during walking can be observed. Note that Figure 5b is Figure 1a. It is obvious that direct comparison of Figure 5b which is the MoCap trajectory before the mapping and Figure 1b which is the IMU-based trajectory is not possible. Instead, Figure 1b can be compared with Figure 5c which is the MoCap trajectory after the mapping, to evaluate the accuracy of IMU-based trajectory estimation.

**Figure 6:**
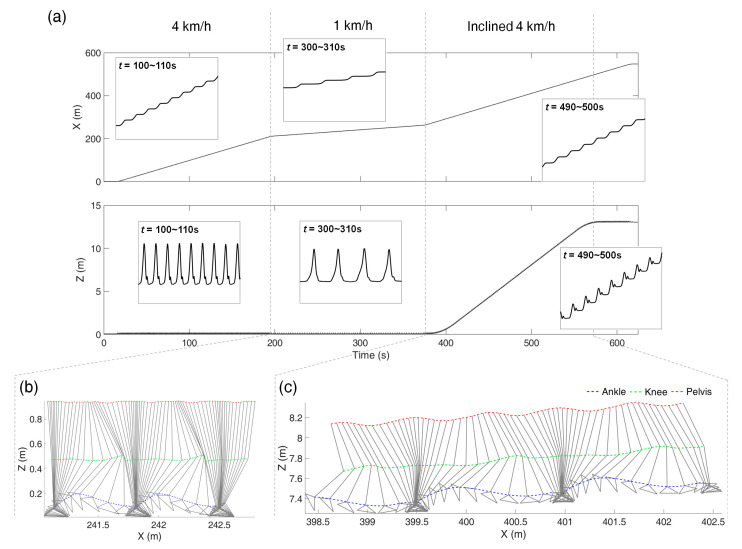
Mapping results from Trial 6 of the variable-speed tests with various inclinations: (**a**) shows the *x*- and *z*-axes ankle marker trajectories with respect to time, (**b**,**c**) show marker trajectories on the sagittal plane after the mapping, during 1 km/h walking without inclination (*t* = 300–306 s) and during 4 km/h with the inclination of 4° (*t* = 490–493 s), respectively.

Figure 6 is the mapping result of Trial 6 with the variable-speed walking test with various inclinations as an example. Figure 6a shows the *x*- and *z*-axes trajectory mapping results of the ankle marker over time, and Figure 6b,c show the marker trajectories on the *x*–*z* plane after the overground mapping, for 1 km/h without inclination and for 4 km/h with inclination, respectively. Comparing the walking at 4 km/h with and without inclination, the gradient of the *z*-position increases rapidly during the inclined period. In the enlarged graph of the time range of 490–500 s, the *z*-axis displacement occurs only in the swing phase.

## 4. Discussion and Conclusions

This paper presented the T2OG mapping method of optical marker trajectories for continuous gait analysis by adopting the simple concept of virtual origin as follows. As the treadmill belt moves backward, the position of the virtual origin attached onto the belt moves backward. The position vector from the backward moving virtual origin to a targeted marker within a limited walking volume is the same as the position vector from the fixed origin to the forward moving marker over the ground. After the T2OG mapping, the marker trajectory during the treadmill walking on a track belt rotating in an infinite orbit is not confined to a limited treadmill walking volume but extends on an unfolded belt, which can be considered the overground.

As mentioned earlier, the IMU-based trajectory data do not stay in the treadmill-located limited space even if the data measurements are performed on a treadmill. Instead, the IMU-based treadmill walking data proceed forward like the overground walking data. Accordingly, direct comparison of IMU-based trajectory and MoCap-based trajectory is not possible without the mapping process. This necessity is our particular interest as we are working on IMU-based wearable gait tracking systems, for which accuracy should be verified using the MoCap measurement. To our knowledge, nevertheless, research on this has not been conducted yet. Considering the wide applicability and popularity of the IMU as a wearable motion sensor, the proposed method is highly valuable in that it provides an accurate reference for the treadmill-based gait analysis with IMU signals.

As a proof-of-concept study, only one subject performed the pedestrian walking test and the meaning of the figures in the validation results may be limited. Further validations using multiple subjects are warranted to generalize the proposed methodology. However, this study demonstrated the feasibility of the T2OG mapping of marker trajectory for treadmill-based continuous gait analysis.

The accuracy of the proposed method was verified from various treadmill walking tests, which showed the TTD error rate of 0.32% on average. Note that most of the errors in the T2OG mapping are caused by an accumulation of the very small errors from the MoCap system, by considering that the TTD error rates shown in Table 1 and Table 2 are close to the error rates from the linear scale. The proposed method estimates the displacement of the track belt by continuously integrating the marker displacement in the same section of the marker chain. Therefore, if there is a tiny measurement error in the marker position from the MoCap system, the tiny error is continuously accumulated in the estimated track belt displacement. Moreover, as the travel distance increases, the effect of MoCap calibration on the mapping accuracy becomes larger. In this regard, when using the proposed method, it is necessary to pay careful attention to the calibration to minimize estimation errors. While the proposed method provides a gold standard reference based on the MoCap-based trajectory data for the accuracy evaluation of the IMU-based trajectory data, securing a reference to verify the accuracy of the proposed method is a separate task. Further research is needed in this issue.

With the proposed method, it is possible (i) to observe the change in physical quantity visually during the treadmill walking and (ii) to obtain overground-mapped gait data for evaluating the accuracy of the IMU-based trajectory estimation. Furthermore, the proposed method can be linked to various potential applications for Industry 4.0 and rehabilitation robotics by improving the dynamics of human-robot collaboration [39,40,41,42,43,44]. For example, using the proposed approach, the collaborating robot working as a rehabilitation robot in the area of physiotherapy will be able to better track the day-to-day and real-time progress of a stroke patient practicing to walk again by walking on a treadmill during the rehabilitation phase.

## Figures and Tables

**Table 1:** Total travel displacement (TTD) errors of the tests without pedestrian walking (mean ± standard deviation).

Speed	Reference	Error	Error Rate
(**a**) Camera setting 1			
2 km/h	18,080.3 cm	36.2 ± 0.5 cm	0.20 ± 0.003%
4 km/h	36,239.7 cm	66.0 ± 0.3 cm	0.18 ± 0.001%
6 km/h	54,412.0 cm	101.2 ± 0.5 cm	0.19 ± 0.001%
(**b**) Camera setting 2			
2 km/h	18,080.0 cm	183.2 ± 1.0 cm	1.01 ± 0.005%
4 km/h	36,238.1 cm	365.2 ± 1.47 cm	1.01 ± 0.004%
6 km/h	54,417.9 cm	542.1 ± 2.47 cm	1.00 ± 0.005%

**Table 2:** TTD errors of the tests with pedestrian walking (unit: cm).

(**a**) Constant-speed walking without inclination				
	**Trial 1**	**Trial 2**	**Trial 3**	**Average**
Reference	35,198.7	35,177.7	35,179.6	-
Proposed	35,311.4	35,287.5	35,293.0	-
Error	112.7	109.8	113.4	111.9
Error rate	0.32%	0.31%	0.32%	0.32%
(**b**) Variable-speed walking with various inclinations				
	**Trial 4**	**Trial 5**	**Trial 6**	**Average**
Reference	54,596.4	54,582.1	54,548.0	-
Proposed	54,763.4	54,749.8	54,715.2	-
Error	169.8	167.0	167.7	167.3
Error rate	0.31%	0.31%	0.31%	0.31%

## Data Availability

The data presented in this study are available on request from the corresponding author.
